# Process, structural, and outcome quality indicators to support perioperative opioid stewardship: a rapid review

**DOI:** 10.1186/s13741-023-00312-4

**Published:** 2023-07-10

**Authors:** C. Thomas, M. Ayres, K. Pye, D. Yassin, S. J. Howell, S. Alderson

**Affiliations:** 1 Department of Anaesthesia, St. James’ University Hospital, Leeds Teaching Hospitals NHS Trust, Leeds, LS9 7TF UK; 2 Department of Anaesthesia, Leeds Teaching Hospitals NHS Trust, Leeds, UK; 3 Leeds Institute of Health Research, University of Leeds, Leeds, UK; 4 Primary Care, Leeds Institute of Health Sciences, University of Leeds, Leeds, UK

**Keywords:** Analgesics, Opioids, Opioid stewardship, Quality indicators, Colorectal cancer surgery

## Abstract

Opioids are effective analgesics but can cause harm. Opioid stewardship is key to ensuring that opioids are used effectively and safely. There is no agreed set of quality indicators relating to the use of opioids perioperatively. This work is part of the Yorkshire Cancer Research Bowel Cancer Quality Improvement programme and aims to develop useful quality indicators for the improvement of care and patient outcomes at all stages of the perioperative journey.

A rapid review was performed to identify original research and reviews in which quality indicators for perioperative opioid use are described. A data tool was developed to enable reliable and reproducible extraction of opioid quality indicators.

A review of 628 abstracts and 118 full-text publications was undertaken. Opioid quality indicators were identified from 47 full-text publications. In total, 128 structure, process and outcome quality indicators were extracted. Duplicates were merged, with the final extraction of 24 discrete indicators. These indicators are based on five topics: patient education, clinician education, pre-operative optimization, procedure, and patient-specific prescribing and de-prescribing and opioid-related adverse drug events.

The quality indicators are presented as a toolkit to contribute to practical opioid stewardship. Process indicators were most commonly identified and contribute most to quality improvement. Fewer quality indicators relating to intraoperative and immediate recovery stages of the patient journey were identified. An expert clinician panel will be convened to agree which of the quality indicators identified will be most valuable in our region for the management of patients undergoing surgery for bowel cancer.

**Supplementary Information:**

The online version contains supplementary material available at 10.1186/s13741-023-00312-4.

## Background

Inappropriate opioid prescribing is an internationally recognized threat to population health and a pressing challenge for healthcare services (Kiang et al. 2020; Curtis et al. 2019; Degenhardt et al. 2019). The North American ‘opioid crisis’ continues, with an ongoing rise in opioid-related mortality, initially due to prescription opioids and more recently illicit heroin and fentanyl use (Berterame et al. 2016). Despite increased awareness of risks and opioid abuse, prescription opioid use remains historically high in both North America and Europe (Jani et al. 2020; Schieber et al. 2019; Verhamme and Bohnen 2019; Lancet 2022). Inappropriate prescribing following surgery is increasingly recognized as a contributor to the problem. Opioids are effective analgesics for managing acute pain following surgical trauma (Small and Laycock 2020) and were increasingly used in longer and higher doses following the publication of guidelines on post-operative pain management (Ballantyne et al. 2016). However, opioids also have significant adverse effects, including sedation, constipation, nausea, and confusion. Long-term use can lead to tolerance, dependence, hyperalgesia, addiction and increased mortality (Colvin et al. 2019). There is increasing attention being paid to the role of postoperative opioids in slowing recovery from surgery and contributing to long-term opioid use (Glare et al. 2019; Levy et al. 2021; Daliya et al. 2021). Enhanced recovery after surgery (ERAS) programmes often include the provision of multi-modal analgesia to promote faster recovery with fewer complications. Whilst short-term opioid use provides effective relief from acute pain following surgery, it is increasingly recognized that the perioperative period is a time when longer-term opioid usage may begin. Effective opioid stewardship in the perioperative period is therefore of critical importance.

Improving opioid stewardship in the peri- and post-operative management of patients with bowel cancer has the potential to improve recovery, lead to faster discharge, improve outcomes and most importantly, prevent patient harm. A requirement for an effective opioid stewardship program is the ability to measure the appropriateness of opioid use.

Health care quality indicators are a type of performance measure (Stelfox and Straus 2013) that evaluate aspects of quality of care, without which the monitoring of healthcare quality is impossible (Mainz 2003; Arah et al. 2006). Quality indicators are used to measure the variability in the quality of care, identify potential areas for improvement and can be used to feedback on performance to healthcare teams to change clinical practice. They should be relevant, actionable, reliable, show room for improvement and data collection should be feasible (Ivers et al. 2012; Kelley and Hurst 2006; Fabian and Geppert 2011). Donabedian’s framework (Donabedian 1988) describes quality as a function of three domains: structure, process and outcome. The structure is defined by the attributes of the setting in which care is provided, process by the input of the practitioners working in that system and outcome by the change in health status of the patient.

No quality indicators for perioperative opioid use are currently described in the National Institute for Health and Care Excellence Standards and Indicators library (Standards and Indicators | NICE (accessed 22nd March 2022). A rapid review was performed to identify quality indicators for perioperative opioid stewardship for patients undergoing abdominal surgery for bowel cancer. This is a form of knowledge synthesis that streamlines the process of conducting a traditional systematic review to produce evidence in a rapid resource-efficient manner (Hamel et al. 2021) and has been chosen to allow timely evidence synthesis to inform decision-making (Haby et al. 2016).

The objective of this rapid review was to identify and extract potential quality indicators from the best available evidence on perioperative opioid use in patients undergoing major abdominal surgery for bowel cancer. This approach to the development of actionable quality indicators has been described and applied effectively in other clinical settings (Kallen et al. 2018).

## Methods

Cochrane rapid review methods were followed (Garrity et al. 2021). A systematic literature search of Medline was performed and included all articles available to April 2021. Systematic reviews and primary studies were sought. The types of participants were not restricted and could be individuals, organizations or systems. Search terms are shown in Table 1 and include terms and truncations for quality indicators, opioids, surgery (with potential limitation to colorectal cancer surgery) and development. The search was limited to studies of adult subjects and studies published in English. A manual search was conducted of the reference lists of the selected papers. Searches were conducted between the 1st and the 25th August 2021 and supplementary searches of reference lists were conducted in December 2021. The National Quality Measures Clearinghouse project (2022) was also reviewed for relevant content.

**Table 1:** MEDLINE search strategy

Quality indicator	AND	Opioids	AND	Abdominal surgery/bowel cancer surgery
1. Quality indicator [Mesh] OR 2. Quality criterion OR 3. Quality measure^*^ OR 4. Performance indicator OR 5. Performance measure OR 6. Outcome measure OR 7. Outcome indicator OR 8. Audit OR 9. Outcome assessment [Mesh] OR 10. Process assessment [Mesh]		1. Analgesics, Opioid [Mesh] OR 2. Opioid^*^ OR 3. Stewardship [tw] OR 4. Appropriate opioid use [tw] OR 5. Opioid use		1. Colonic neoplasms [Mesh] OR 2. Colorectal neoplasms [Mesh] OR 3. Intestinal neoplasms [Mesh] OR 4. Bowel cancer OR 5. Laparoscopy [Mesh] OR 6. Digestive system surgical procedures [Mesh] OR 7. Colectomy [Mesh] OR 7. Bowel cancer surgery OR 9. Abdominal surgery

^*^Truncation symbol = different words/terms can be searched for (singular/plural/conjugations)

Limited to English language and adults

The initial search identified 588 abstracts. These results were imported into Rayyan (http://rayyan.qcri.org/) (Ouzzani et al. 2016) a free web tool used to facilitate the screening and selection of studies for systematic and scoping reviews.

Three members of the project team (MA, KP, DY) screened all titles and abstracts for inclusion. Duplicate studies, case reports, editorials, non-English language studies, quality improvement not concerning opioid use, abdominal or colorectal surgery, or performance measures and quality improvement in specific subgroups of patients were excluded. Where a decision on inclusion could not be reached, two further team members (CT, SH) reviewed the titles and abstracts. All studies included at this stage underwent full-text review, undertaken by two members of the project team (CT, SH). MA, KP, and DY accessed the full-text articles of all included papers, which were uploaded and accessed using Rayyan. The extraction of quality indicators from the full texts was undertaken by CT and SH. All those included were papers from which quality indicators could be extracted. A quality indicator extraction tool was developed in advance of data extraction, with potential indicators categorized to the stage of perioperative care they relate to (Supplementary materials 1) to enable reproducible results. Finally, a full list of potential indicators was composed, in which indicators were rephrased where needed and duplicate indicators removed. Reporting has been guided by the PRISMA extension for scoping reviews (PRISMA-ScR) (Extension and for Scoping Reviews (PRISMA-ScR): Checklist and Explanation | The EQUATOR Network (equator-network.org) (accessed 22nd March 2022).

## Results

**Fig. 1:**
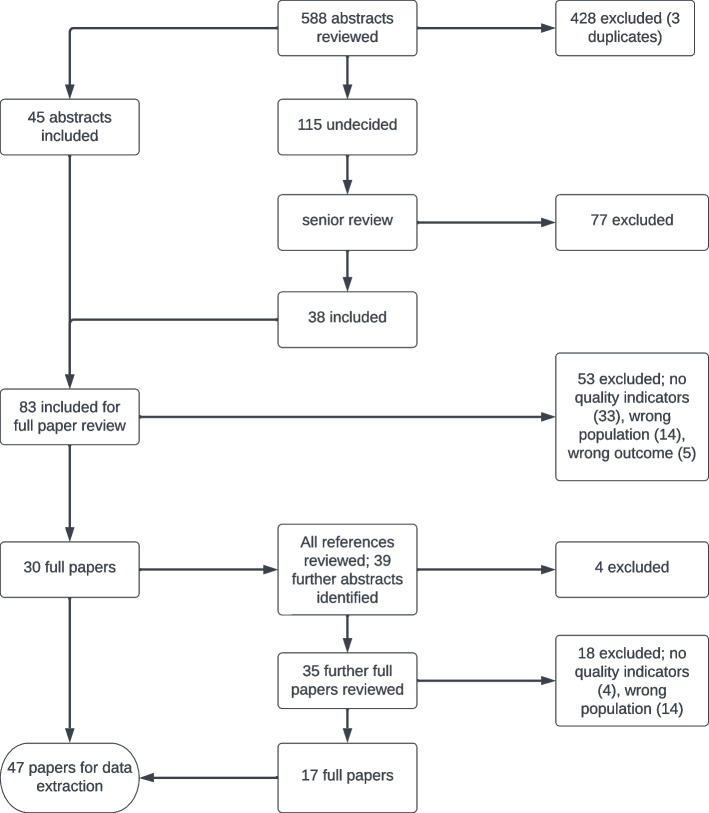
Study selection flowchart

Five hundred eighty-eight publications were identified by the literature search. Three duplicates and a further 425 abstracts were removed as they did not meet inclusion criteria. Eighty-three of these papers were included for full-text review, of which 53 were excluded because they reported on another outcome or population, or because the paper did not include quality indicators. Thirty papers were included, the references of which were reviewed. A further 35 full-text papers were reviewed, of which 17 were included. The study selection flowchart (Fig. 1) details this process. In total, 47 papers were identified from which quality indicators were extracted. Review of the quality indicators clearinghouse did not yield results.

The characteristics (study design and numbers of participants) of the included papers are shown in Supplementary Materials 2.

One hundred twenty-eight quality indicators from 47 papers were extracted, with some papers describing several indicators. See Supplementary materials 1 for full details of all raw extracted quality indicators. Duplicates were removed, leading to the identification of 24 discrete indicators. Table 2 shows the numbers of discrete quality indicators identified at each stage of the patient journey.

**Table 2:** Numbers of discrete quality indicators identified at each stage of the patient journey

Stage of patient journey	Number of quality indicators	Number of papers	Number of distinct quality indicators
Pre-operative	26	19	7
Intra-operative	13	12	1
Recovery	5	13	2
Post-operative	19	17	4
Discharge	43	17	6
Follow up	22	13	4

Instruments for collecting data on quality indicators, and structural, process, and outcome indicators were collated. These are grouped according to stage in the perioperative journey and are shown in Table 3.

**Table 3:** Structural, process, and outcome quality indicators across the patient perioperative journey

	First author, year of publication	Brief topic of quality indicator	Instruments for collecting data on quality indicators	Structural/process quality indicators	Outcome quality indicators
*Preoperative*	(Bardiau et al. 1999) (Fields et al. 2019) (Lee et al. 2017) (Neuman et al. 2019)	Patient education		Presence of preoperative patient education materials on perioperative pain and pain management, risks of opioids	
	(Bongiovanni et al. 2020) (Hopkins et al. 2020)	Staff education		Presence of multi-professional education materials for staff on opioid stewardship and need for multimodal analgesia	
	(Brat et al. 2018) (Cron et al. 2017) (Fields et al. 2019) (Gan et al. 2020) (Hilliard et al. 2018) (Truong et al. 2019)	Preoperative identification and optimization for patients with opioid tolerance	Opioid tolerant definition: if opioids used for more than 7 days in the 60 days prior to surgery/any opioid use in 12/12 prior to surgery, any opioid medication on admission meds list	System to identify preoperative opioid use in elective population Specialist pain referral pathway to enable opioid weaning and perioperative analgesic planning for preoperative optimization in opioid tolerant population	
	(Brummett et al. 2017) (Clarke et al. 2014) (Fields et al. 2019) (Jiang et al. 2017) (Lee et al. 2017) (Macintyre et al. 2014) (Stafford et al. 2018)	Identification of patients at greatest risk of persistent postoperative opioid use (PPOU)	Opioid Risk Tool (ORT), Screener for Opioid Assessment and Patients with Pain (SOAPP) and Brief Risk Interview (BRI) may be of use in acute pain setting	Presence of screening tool to identify risk factors for persistent postoperative opioid use (PPOU) defined as use of opioids at 90–180 days postoperatively Identified prevalence of PPOU risk	
	(Minkowitz et al. 2014)	Identification of patients at risk of opioid-related adverse drug events (ORADEs)		Presence of screening tool to identify preoperatively those at greater risk of postoperative opioid-related adverse drug events (ORADEs)	
	(Felling et al. 2018) (Yap et al. 2020)	Use of multimodal analgesia		Presence of protocol to reduce perioperative opioid use with preoperative multimodal analgesia	
	(Lee et al. 2017) (Macintyre et al. 2014)	Concept of ‘universal precautions’ in the use of perioperative opioids		Adoption of ‘universal precautions’ when initiating perioperative opioids	
*Intraoperative*	(Bardiau et al. 1999) (Brandal et al. 2017) (Cheung et al. 2009) (Felling et al. 2018) (Keller et al. 2019) (Mujukian et al. 2020), (Neuman et al. 2019) (Stafford et al. 2018) (Thiele et al. 2015) (Truong et al. 2019) (Wick et al. 2017) (Yap et al. 2020)	Use of multimodal analgesia		Presence of opioid-sparing protocol for intra operative use including minimally invasive surgery, regional blocks and multimodal analgesia Adherence to intra-operative opioid sparing protocol	
*Recovery*	(Fields et al. 2019)	Identification of patients at greatest risk of PPOU		Presence of review/re-screen with new risk factors for PPOU including formation of a stoma	
	(Bardiau et al. 1999) (Brandal et al. 2017) (Cheung et al. 2009) (Felling et al. 2018) (Keller et al. 2019) (Mujukian et al. 2020) (Neuman et al. 2019) (Stafford et al. 2018) (Thiele et al. 2015) (Truong et al. 2019) (Wick et al. 2017) (Yap et al. 2020)	Use of multimodal analgesia		Presence of opioid-sparing protocol for recovery/immediate postoperative use including regional blocks and multimodal analgesia Adherence to recovery/immediate postoperative opioid sparing protocol	
*Postoperative*	(Bardiau et al. 1999) (Brandal et al. 2017)	Access to acute pain service		Availability of an acute pain service Delivery of a daily pain review	
	(Bardiau et al. 1999) (Brandal et al. 2017) (Cheung et al. 2009) (Felling et al. 2018) (Gan et al. 2015) (Keller et al. 2019) (Mujukian et al. 2020) (Neuman et al. 2019) (Stafford et al. 2018) (Thiele et al. 2015) (Truong et al. 2019) (Wick et al. 2017) (Yap et al. 2020)	Use of multimodal analgesia		Presence of opioid-sparing protocol for postoperative use including regional blocks and multimodal analgesia Adherence to postoperative opioid sparing protocol	Rate of postoperative ileus
	(Keller et al. 2019) (Kessler et al. 2013) (Lee et al. 2010) (Oderda et al. 2013) (Tsui et al. 1996)	Presence of ORADEs	Scoring of frequency, severity, and distress of opioid-related side effects as 0 to 60 on the Perioperative Opioid-related Symptom Distress scale	Presence of review for ORADEs	Rate of ORADEs, severity of ORADEs detected, impact of ORADEs on length of stay
	(Greco et al. 2014) (Neuman et al. 2019) (Syrowatka et al. 2021)	Protocolized opioid prescribing in hospital		Procedure-specific protocol for use of in-hospital opioids, promoting avoidance of long acting opioids Electronic clinical quality measure (eCQM) to assess potentially inappropriate high dose postoperative opioid prescribing practices, e.g., an average daily dose ≥ 90 MME for the duration of postoperative opioid prescription in preoperatively opioid naïve patients	
***Discharge***	(Brandal et al. 2017) (Bromberg et al. 2021) (Chen et al. 2018) (Fields et al. 2019) (Fujii et al. 2018) (Hill et al. 2017) (Hill et al. 2018) (Hopkins et al. 2020) (Lee et al. 2017) (Macintyre et al. 2014) (Neuman et al. 2019) (Pruitt et al. 2020) (Thiels et al. 2017) (Wang et al. 2021) (Wick et al. 2017)	Protocolized opioid prescribing on discharge	Procedure-specific MME centiles to reduce inter-prescriber variation	Presence of a patient group specific guideline or algorithm for discharge opioid prescribing, opioid use in 24 h prior to discharge to guide opioids prescribed on discharge aiming at prescribing the lowest dose opioid possible for the shortest duration Procedure specific post op prescribing guidelines to provide enough doses to cover 75% of patients Procedure specific prescribing limits built into electronic patient record Procedure-specific mean discharge MME prescribed	Total milligram of morphine equivalents (MME) consumed during 24 h prior to discharge Opioid present on hospital discharge prescription Frequency of slow-release opioids prescribed on discharge Frequency of immediate-release opioids prescribed on discharge Non-opioid adjuvant analgesia present on discharge prescription
	(Brandal et al. 2017) (Wang et al. 2021)	Review of inpatient opioid use		Presence of recording tool for opioids used during inpatient stay	Total milligram of morphine equivalents (MME) consumed during hospital stay Procedure specific mean daily inpatient MME used
	(Fields et al. 2019) (Hoang et al. 2020)	Identification of patients at greatest risk of PPOU	Use > 90th centile MME opioids, or equivalent of over 50 5 mg oxycodone prescribed at discharge as risk factor/flag for PPOU		
	(Hopkins et al. 2020) (Macintyre et al. 2014)	Opioid de-escalation and tapering		Presence of a de-escalation plan for opioids prescribed on discharge Use of ‘reverse pain ladder’ to guide de-escalation Pain management plan and tapering strategies clearly communicated to primary care team in a timely manner	
	(Bartels et al. 2016) (Fujii et al. 2018) (Lee et al. 2017) (Hill et al. 2017) (Macintyre et al. 2014) (Neuman et al. 2019)	Patient education		Provision of patient education on safe storage and disposal of unused opioids and avoidance of opioid diversion Opioid-specific discharge advice, e.g., do not drive for up to 4 weeks until opioid dose is stable	
	(Macintyre et al. 2014)	Identification of patients at risk of ORADEs		Identify those at risk of ORADEs when prescribing opioids for use at home. Male, obese, over 65, greater comorbidities, pre-op opioid use, concurrent sedative medication use.	
***Follow up***	(Agarwal et al. 2021) (Bartels et al. 2016) (Bromberg et al. 2021) (Howard et al. 2019) (Meyer et al. 2021) (Pruitt et al. 2020) (Roughead et al. 2019)	Review of opioids prescribed v used	MME prescribed and consumed	Presence of process to assess opioids prescribed v opioids used following surgical procedures to allow tailoring of opioid prescriptions to need for a patient group/specific procedure reduce unused opioid in the community	Post op prescription considered to have been given if opioids dispensed between 2–7 days following discharge
	(Brat et al. 2018) (Clarke et al. 2014) (Fields et al. 2019) (Hill et al. 2018) (Pullman et al. 2021) (Roughead et al. 2019)	Identification of patients at greatest risk of or with PPOU	In primary care, detection of opioid misuse/PPOU after discharge, defined as at least one of the ICD-9 diagnosis code of opioid dependence, abuse, or overdose	Hospital analgesic policies include strategies to support post-discharge assessment and follow-up of patients at risk of becoming chronic opioid users	New or repeat opioid prescriptions within 30 days of discharge Use of higher dosage of opioids at any time (> 50–60 MME) PPOU: ongoing opioid use at 90–180 days post-discharge Incidence of opioid-related re-admissions Time to opioid cessation: a period without an opioid prescription equivalent to three times the estimated supply duration in preoperatively opioid naïve patients
	(Pruitt et al. 2020)	Staff education		Staff education: prescribers sent quarterly reports on their prescribing v guidelines	
	(Macintyre et al. 2014)	Management of those with PPOU		Presence of plan/protocol if opioid abuse or misuse is detected	

The quality indicators identified which could be grouped into five topics: patient education, staff education, preoperative patient optimization, patient and procedure-specific prescribing and deprescribing and opioid-related adverse drug events (ORADEs) and are shown in Table 4. Full details of the quality indicator topics are shown in Supplementary materials 3.

**Table 4:** Full list of proposed quality indicators

Theme	Proposed quality indicators
Patient education	The site provides and delivers patient education materials in the preoperative period which cover expectations of perioperative pain and pain management options including the risks and benefits of opioids
	The site provides and delivers patient education materials at discharge which cover the provision of patient education on safe storage and disposal of unused opioids in the community, the requirement to avoid opioid diversion, and opioid specific discharge advice, e.g., DVLA requirements
Staff education	The site provides and delivers multi-professional education materials on opioid stewardship
	The site provides and delivers multi-professional education materials on the provision of multimodal analgesia at all stages of the patient journey starting in the preoperative setting
	Percentage of prescribers who receive regular reports comparing their prescribing to hospital guidelines
	The site provides and delivers educational materials on the need for a clear discharge pain management plan and tapering strategy
Preoperative patient optimization	The presence of a system to identify opioid tolerance preoperatively, defined as opioids used for 7 days or fewer in the 60 days prior to surgery.
	The provision of a specialist pain service and referral pathway to enable opioid weaning and patient-specific analgesic planning for preoperative optimization for patients with opioid tolerance
	The site uses a preoperative screening tool to identify patients with risk factors for persistent postoperative opioid use (PPOU)
Patient and procedure-specific prescribing and deprescribing	The site has an acute pain service with the ability to provide a daily pain review
	The electronic record is used as a means to detect or highlight potentially inappropriate high-dose postoperative opioid prescriptions
	Review takes place to evaluate the procedure-specific mean daily inpatient MME used
	Use of higher dosage of opioids (> 50–60 MME per day) at any time during the perioperative journey is used as a flag for further review
	The site has a perioperative analgesia protocol which includes regional blocks and multimodal analgesia
	The presence of procedure-specific protocols for use of in-patient opioids specifically promoting the avoidance of long-acting opioids
	The presence of a review postoperatively seeking new risk factors for PPOU identified including, e.g., formation of a stoma
	The percentage of those who are still using opioids at 90–180 days postoperatively (where the denominator is patients undergoing major surgery for bowel cancer)
	The use of protocolized opioid prescribing for hospital discharge:
	The site has a system to guide prescribing
	The site has a system to allow the review of the procedure-specific mean discharge opioids prescribed for a particular patient group
	The site has a patient group-specific guideline or algorithm to guide discharge opioid prescribing
	The electronic record is used to enable procedure-specific prescribing limits
	Procedure-specific postoperative prescribing guidelines are used to provide enough doses at discharge to cover 75% of patients (where the denominator is all patients undergoing that procedure)
	The site has a system in place to allow the discharge pain management plan and tapering strategy to be clearly communicated to primary care team in a timely manner The opioid requirement, e.g., total consumed during the 24 h prior to discharge is used as a guide for opioids prescribed on discharge
	The presence of a review process for opioid prescription at discharge, where the denominator is all patients discharged having had a major surgery for bowel cancer:
	The frequency of any opioids prescribed on hospital discharge
	The frequency of slow-release opioid prescription on discharge
	The frequency of immediate-release opioid prescription on discharge
	The frequency of non-opioid adjuvant analgesia prescription on discharge
	The presence of a protocol to guide de-escalation plan for opioids prescribed on discharge
	Protocolized use of the ‘reverse pain ladder’ to guide de-escalation
	Pain management plan and tapering strategy clearly communicated to the primary care team in a timely manner
	The presence of a process to assess opioids prescribed versus opioids actually used following surgical procedures to allow tailoring of opioid prescriptions to need for a patient group/specific procedure
	The presence of patient screening for risk of PPOU at discharge
	Follow up for patients at greatest risk of persistent postoperative opioid use
	The presence of a system to detect new or repeat opioid prescriptions given within 30 days of discharge
	The presence of a protocol or clear plan to follow if opioid abuse or misuse is detected
Opioid-related adverse drug events (ORADEs)	The site uses a preoperative screening tool to identify patients at greatest risk of postoperative opioid-related adverse drug events (ORADEs). Documented risk factors are those who are male, obese, over 65, with comorbidities, a history of preoperative opioid use and those concurrently using sedative medication.
	The site has a system in place to detect ORADEs among postoperative inpatients
	There is a system in place to detect ORADEs in the community setting following discharge

## Discussion

Opioids are highly effective analgesics but can cause harm and there is now increasing concern about their perioperative use. A number of contributing problems have been identified. Opioid tolerance preoperatively is a risk factor for poorer outcome (Cron et al. 2017; Gan et al. 2020; Gan et al. 2015; Kessler et al. 2013). When opioids are used by either opioid-naïve or opioid tolerant patients, they are put at risk of opioid-related adverse drug events (ORADEs) (Macintyre et al. 2014; Minkowitz et al. 2014; Keller et al. 2019; Kessler et al. 2013; Oderda et al. 2013), and opioid use is associated with postoperative complications and increased length of stay (Cron et al. 2017; Gan et al. 2020; Gan et al. 2015). Additionally, the perioperative period has been identified as a period of risk for the development of chronic opioid use (Lee et al. 2017; Brummett et al. 2017; Clarke et al. 2014; Macintyre et al. 2014; Roughead et al. 2019; Srivastava et al. 2021). At discharge, opioid prescriptions in excess of requirements are widely reported (Neuman et al. 2019; Bromberg et al. 2021; Hill et al. 2017; Hill et al. 2018; Pruitt et al. 2020; Bartels et al. 2016; Agarwal et al. 2021; Howard et al. 2019; Meyer et al. 2021) and opioids initially used for short-term pain relief can become part of repeat prescriptions following hospital discharge. Poor practice around safe storage and disposal of opioids following discharge contributes to increased opioid in the community with the potential for opioid diversion (Fujii et al. 2018; Hill et al. 2017; Bartels et al. 2016). These factors contribute to the development of persistent postoperative opioid use (PPOU) with the increased potential for ORADEs in the community following discharge.

Effective opioid stewardship is therefore an important part of the provision of opioids in the perioperative period, and a need to improve has been identified (Srivastava et al. 2021). Quality indicators are used to monitor and improve quality in healthcare (Stelfox and Straus 2013; Mainz 2003; Fabian and Geppert 2011; Donabedian 1988; Rademakers et al. 2011). Good quality indicators are based on the best available evidence, should be highly specific and sensitive, with the integration of best clinical evidence, clinical expertise, and patient values. No quality indicators for perioperative opioid stewardship currently exist. The review of the supporting evidence base is required to enable the development of a practical set of reliable quality indicators (Stelfox and Straus 2013).

### Extracted quality indicators

Our review identified indicators relating to five key topics during the perioperative patient journey. These five topics are patient education, staff education, preoperative patient optimization, patient and procedure-specific prescribing and deprescribing and opioid-related adverse drug events. All five topics include structure, process, and outcome quality indicators (Table 4).

### Definitions and comparisons

Varying definitions used in the literature have emerged from this review and consideration of these when discussing quality indicators is useful. Persistent perioperative opioid use is frequently described as the ongoing use of opioids at 90–180 days postoperatively (Fields et al. 2019; Lee et al. 2017; Clarke et al. 2014; Roughead et al. 2019; Pullman et al. 2021). Opioid tolerance is variably described as being present if a patient has used opioids for more than 7 days in the 60 days prior to surgery, any opioid use in 12 months prior to surgery or any opioid on the admission medication list (Fields et al. 2019; Brat et al. 2018; Cron et al. 2017; Gan et al. 2020; Hilliard et al. 2018; Truong et al. 2019). Milligrams of morphine equivalents (MME) or oral morphine equivalents (OME) are the most widely used methods to describe and compare opioid use. When reviewing postoperative patients in the community, the postoperative prescription can be considered to have been used if the prescribed opioids are dispensed between 2 and 7 days following discharge (Roughead et al. 2019). The detection of opioid misuse or PPOU after discharge is defined as at least one of the ICD-9 diagnosis code of opioid dependence, abuse or overdose (Brat et al. 2018). When reviewing the time to opioid cessation, a suggested definition is a period without an opioid prescription equivalent to three times the estimated supply duration in preoperatively opioid naïve patients (Roughead et al. 2019).

### Data collection tools

Instruments to collect data for quality indicators are also reported although none have been specifically developed for postoperative opioid use. The Opioid Risk Tool (ORT), Screener for Opioid Assessment and Patients with Pain (SOAPP), and Brief Risk Interview (BRI) have been proposed for use in perioperative practice when screening patients preoperatively for risk of PPOU (Macintyre et al. 2014). The frequency, severity, and distress caused by opioid-related side effects can be scored as 0 to 60 on the Perioperative Opioid-related Symptom Distress scale and has been reported as a tool to assess ORADEs (Lee et al. 2010).

### Addressing the gaps

More process quality indicators than structure or outcome quality indicators are described in the literature. However, the factors which are reported to make the greatest difference to a patient’s assessment of healthcare quality are process-related and process quality indicators are especially useful to consider when quality improvement is desired (Rademakers et al. 2011). Fewer quality indicators concern the intraoperative and immediate recovery period. The impact of specific changes in practice on long-term outcomes remains unclear, and our rapid review of quality indicators will enable rigorous studies of the implementation and impact of interventions to improve opiate stewardship in the perioperative period.

### Algorithms and electronic systems

The screening of patients for potential opioid tolerance, future likelihood of PPOU, and patient-group-specific prescribing with limits on the type, dose, and duration of opioid prescription may be best undertaken with the use of algorithms and the development in machine learning (Ellis et al. 2019). Electronic records and prescribing (which are already well-embedded in primary care) are now used increasingly in hospital clinical practice and this may present a good opportunity to develop patient-or patient-group-specific guidelines for opioid prescribing with limits and alerts if there is deviation from agreed protocols.

### Limitations

Limitations of this work include those relating to rapid review methodology. This is a relatively recently developed form of knowledge synthesis, and while valid (Garrity et al. 2021), is less comprehensive than a systematic review. Most of the studies included are retrospective cohort studies, and most originate using data from patients in a different healthcare systems (often from the USA). The characteristics of papers are reported (Supplementary Materials 2) but an assessment of risk of bias was not undertaken. This work has been done to drive improvement in outcomes for patients undergoing bowel cancer and this may limit its applicability to a wider perioperative population.

### Conclusion and future work

The concept of ‘universal precautions’ have been suggested as being applicable to the prescribing and administration of opioids in the perioperative period (Lee et al. 2017; Macintyre et al. 2014) and encompass strategies at each stage of a patient’s perioperative journey to ensure that the lowest dosage, shortest acting opioids are used for the shortest possible time, while ensuring good analgesia and patient satisfaction. This will be used as an underpinning principle for our ongoing work.

This project forms part of the wider YCRBCIP program for use in the improvement of outcomes for patients with bowel cancer undergoing surgery. We have identified a set of quality indicators which may help to improve quality of care for patients undergoing major abdominal surgery for bowel cancer who receive perioperative opioids. We will now integrate the extracted quality indicators with clinician expertise and patient values to develop a more concise toolkit which providers in our region can use to benchmark and improve quality in the use of perioperative opioids for patients with bowel cancer.

## Supplementary Information

**Additional file 1: Supplementary materials 1.** Extracted quality indicators

**Additional file 2: Supplementary materials 2.** Characteristics of papers

**Additional file 3: Supplementary materials 3.** Quality indicators and themes

## Data Availability

All data generated or analysed during this study are included in this published article [and its supplementary information files].
