# Supplementary material for: Process, structural, and outcome quality indicators to support perioperative opioid stewardship: a rapid review
Source: Perioper Med (Lond). 2023 Jul 10;12:34. doi: 10.1186/s13741-023-00312-4 (PMC10332041; doi:10.1186/s13741-023-00312-4)
Supplement: Supplementary file 1 — Additional file 1: Supplementary materials 1. Extracted quality indicators [file 13741_2023_312_MOESM1_ESM.docx]

|  | **Paper** | **Reference**  **(1^st^ author, year of publication)** | **Pre admission/ pre op** | **Intra**  **op** | **Recovery** | **Post op** | **Discharge** | **Follow up** |
| --- | --- | --- | --- | --- | --- | --- | --- | --- |
| 1 | Patient reported opioid consumption and pain intensity after common orthopaedic and urologic surgical procedures with use of an automated text messaging system | Agarwal 2020 JAMA network |  |  |  |  |  | Text messaging to assess opioids prescribed v opioids used following surgical procedures to tailor opioid prescriptions to need |
| 2 | Effectiveness of an Acute Pain  Service Inception in a General  Hospital | Bardiau, 1999, J Clin Anaesthesia | Pre-op information to patients about duration and severity of pain | Multimodal analgesia |  | Involvement of an Acute Pain Service |  |  |
| 3 | Adopting best practices in post-operative analgesia prescribing in a  safety-net hospital: Residents as a conduit to change | Bongiovanni 2020 American J Surg | Education package delivered to surgical trainees to promote multimodal analgesia on discharge/ highlight importance of opioid stewardship |  |  |  |  |  |
| 4 | Impact of Enhanced Recovery After Surgery and Opioid-  Free Anaesthesia on Opioid Prescriptions at Discharge  From the Hospital: A Historical-Prospective Study | Brandal 2017, Anaesth Analgesia |  | - Presence of ERAS protocol to guide analgesia - Process measures: protocol adherence eg use of multimodal analgesia including epidural, TAP block, IV lidocaine, IV ketamine |  | Daily review by acute pain team | - Presence of an opioid prescription at hospital   discharge   - Presence of a guideline on discharge to guide opioid prescribing - Total morphine equivalents consumed during hospital stay - Total morphine equivalents consumed during 24h prior to discharge |  |
| 5 | Postsurgical prescriptions for opioid naive patients and  association  with overdose and misuse: retrospective cohort study | Brat, 2017, BMJ. | Recognition of pre-admission opioid use. Consider opioid naïve  if opioids used for 7/7 or less in the 60/7 prior to surgery. |  |  |  |  | Look for opioid misuse after discharge (defined as at least one of the ICD-9 (international  classification of diseases, ninth revision) diagnosis  code of opioid dependence, abuse, or overdose)  Record new or repeat opioid prescriptions within 30 days of discharge  Use morphine milligram equivalent (MME) to make comparisons  Duration and dosage cause for concern: ideally opioid use less than 2/52 post op. Still using opioids at 90/7 post op and/or using higher dosage of opioids (>50-60 MME) associated with greater risk of harm. |
| 6 | Assessment of post-operative opioid prescribing practices in a community hospital ambulatory surgical center | Bromberg 2021 J Opioid Management |  |  |  |  |  | Review opioid prescriptions and use of the opioid prescribed at 1/52 post op to allow discharge protocol to be tailored to patient group |
| 7 | Correlation between 24-hour pre discharge opioid use and amount of opioids prescribed at hospital discharge | Chen 2018 JAMA Surg |  |  |  |  | Tailor discharge opioids; If patient does not use opioids in the 24h prior to discharge, consider no opioids on discharge prescription |  |
| 8 | An audit of postoperative intravenous patient-controlled analgesia with  morphine: Evolution over the last decade | Cheung 2009 Eur J Pain |  | Use multimodal analgesia eg IV analgesia, wound infiltration, regional block alongside opioids |  | Use multimodal analgesia eg IV analgesia, wound infiltration, regional block alongside opioids |  |  |
| 9 | Rates and risk factors for prolonged opioid use after  major surgery: population based cohort study | Clarke, 2014 BMJ | Identify risk factors for PPOU in opioid naïve patients:  High risk patient factors: lower fifth of neighbourhood  income, specific comorbidities (diabetes, heart failure,  pulmonary disease), and use of specific drugs preoperatively  (benzodiazepines, SSRIs, ACE inhibitors). |  |  |  |  | Early post-discharge opioid use: define as  one or more prescriptions for opioids within 1 to 90 days after  surgery. Prolonged opioid use after hospital discharge:  define as one or more opioid prescriptions within 1 to 90 days  after surgery along with one or more prescriptions for opioids  within 91 to 180 days after surgery. |
| 10 | Liposomal Bupivacaine Transversus Abdominis  Plane Block Versus Epidural Analgesia in a Colon  and Rectal Surgery Enhanced Recovery Pathway:  A Randomized Clinical Trial | Felling 2018 Dis Colon Rectum | Use of enhanced recovery pathway with multimodal analgesia commencing in pre operative period to reduce opioid use | Use of enhanced recovery pathway with multimodal analgesia commencing in pre operative period to reduce opioid use |  |  |  |  |
| 11 | Predictors of prolonged opioid use following colectomy | Fields 2019 Dis Colon Rectum | Identification of pre op indicators of prolonged opioid use (use at 90-180 days post op): opioid use in 12/12 prior to surgery, ASA 3 or 4.  Review medications, consider pain specialist referral, patient education pre op to reduce post op opioid consumption. |  | Identify those at risk of PPOU: Formation of ostomy |  | Identify those prescribed >90^th^ centile, or equivalent of over 50 5mg oxycodone for opioid dose at discharge as predictor of PPOU  Develop consensus guidelines on discharge opioid prescribing | Monitoring of the post surgical population to detect rate of PPOU: defined as new opioid  prescription 90 to 180 days postoperatively  Identify those at risk of PPOU: readmissions |
| 12 | Impact of postsurgical opioid use and ileus on  economic outcomes in gastrointestinal surgeries | Gan 2015 Current Medical Research and Opinion |  |  |  | Rate of ileus post op (10% in this study):  Patients with ileus received significantly greater morphine equivalent dose than those without (median: 285 vs. 95 mg) and were twice as likely to have post op ileus. |  |  |
| 13 | A Retrospective Review: Patient-Reported  Preoperative Prescription Opioid, Sedative, or  Antidepressant Use Is Associated with Worse  Outcomes in Colorectal Surgery | Gan 2020 Dis Colon Rectum | Assess pre op opioid use and include as component of pre-op risk stratification |  |  |  |  |  |
| 14 | An Unintended Consequence of a New Opioid Legislation | Hoang 2020 Dis Colon Rectum |  |  |  |  | - Review total milligram   morphine equivalents (MMEs) of opioid pain medication  prescribed at discharge.   - Total number of pills prescribed - MMEs of pain medication prescribed in outpatient setting   (Milligram morphine equivalents calculated as 1.5 MMEs per 1mg oxycodone, 1 MME per 1mg  hydrocodone, 4 MMEs per 1mg hydromorphone |  |
| 15 | Educating junior doctors and pharmacists to reduce  discharge prescribing of opioids for surgical patients:  a cluster randomised controlled trial | Hopkins 2020 Medical Journal of Australia | Presence of education package on periop opioids for clinicians and pharmacists |  |  |  | - Number of patients prescribed slow release opioids on discharge - Number discharged without opioids - Documented de-escalation plan at discharge - Prescribed daily dose of slow release opioids as oral morphine equivalents - Immediate release opioids prescribed on discharge - Overall quantity of opioid prescribed at d/c - Non opioid adjuvant analgesia prescribed |  |
| 16 | Association of opioid prescribing with opioid consumption after surgery in Michigan | Howard 2019 JAMA |  |  |  |  |  | Review opioids prescribed with patient reported opioid consumption after discharge to match prescription to need in the post surgical population and reduce unused opioids in the community  Median OME prescribed and consumed used as marker |
| 17 | Chronic opioid usage in surgical patients in a large academic centre | Jiang 2017 Ann Surg | Risk factors for chronic opioid use identified: Females, BMI not in normal range, under multiple surgical specialties, age 50-59, African-American ethnicity |  |  |  |  |  |
| 18 | Opioid-free colorectal surgery: a method to improve patient and financial outcomes in surgery | Keller 2019 Surgical Endoscopy |  | Use of ERAS protocols including laparoscopic surgery whenever possible to reduce opioid requirement |  | Look for rates of opioid related adverse events (ORADE): respiratory, GI, CNS, GU, other |  |  |
| 19 | The costs and benefits of extending the role of the acute pain service on clinical outcomes after major elective surgery | Lee 2010 Anaesthesia Analgesia |  |  |  | Scoring of frequency, severity, and distress of  opioid-related side effects as 0 to 60 on the perioperative opioid-related  symptom distress scale |  |  |
| 20 | Opioid Consumption Patterns After Anorectal  Operations: Development of an Institutional  Prescribing Guideline | Meyer 2020 Dis Colon Rectum |  |  |  |  |  | Review on a procedure specific basis opioids consumed v prescribed to tailor discharge prescriptions |
| 21 | A Standardized Multimodal Analgesia Protocol Reduces  Perioperative Opioid Use in Minimally Invasive Colorectal Surgery | Mujukian 2019 Journal of Gastrointestinal Surgery |  | Multi modal intra op protocol for minimally invasive colorectal surgery to reduce opioid requirement in first 48 h post op |  |  |  |  |
| 22 | Effect of opioid-related adverse events on outcomes in selected surgical patients | Oderda 2013 Journal of Pain and Palliative Care Pharmacotherapy |  |  |  | Rate of ORADEs developed during hospital stay. Recognition and scoring of ORADEs. Gastrointestinal effects reported: nausea, vomiting, constipation,  central nervous system effects:  excessive somnolence, dizziness, and adverse  effects on cognitive function, psychomotor coor-  dination, balance, and alertness. Pruritus, urinary retention, respiratory depression. Impact of ORADEs on length of stay |  |  |
| 23 | Implementation of a Quality Improvement initiative to decrease opioid prescribing in general surgery | Pruitt 2019 Journal of Surgical Research |  |  |  |  | Morphine Mg equivalents (MME) to compare prescriptions.  Procedure specific post op prescribing guidelines to provide enough doses to cover 75% of patients  Procedure specific prescribing limits built into electronic patient record | Recording opioids at post op 2 weeks to identify prescribed v opioids consumed post op. Providing surgeons with this data reduced prescribing.  Prescribers sent quarterly reports on their prescribing v guidelines |
| 24 | Development and alpha testing of specifications for a prolonged opioid prescribing electronic clinical quality measure (eCQM) | Pullman 2021 AMIA Annual Symposium Proceedings |  |  |  |  |  | ‘Potential Opioid Overuse’ examines  percentage of patients who receive opioid  prescriptions  for 90 days or  longer  with a  n average  daily dosage  exceeding  90 morphine milligram equivale  nts for  management of  chronic non-cancer pain  This  eCQM measures  percentage  of patients prescribed opioids beyond 42 days  after surgery, therefore meeting criteria for prolonged prescribing |
| 25 | Persistence with opioids post discharge  from hospitalisation for surgery in  Australian adults: a retrospective  cohort  study | Roughhead 2019 BMJ Open |  |  |  |  |  | Retrospective review of data; post op prescription considered to have been given if opioids dispensed between 2-7 days post op  Time to opioid cessation defined as a period without an  opioid prescription equivalent to three times the  estimated supply duration in preoperatively opioid naïve patients.  Chronic opioid users defined as preoperatively opioid naïve patients who continued  taking opioids for greater than 90 days post discharge.  Hospital analgesic policies include strategies to support post-discharge assessment and follow-up  of patients at risk of becoming chronic opioid users |
| 26 | Re-tooling an Existing Clinical Quality Measure for Chronic Opioid Use to an Electronic Clinical Quality Measure (eCQM) for Post-Operative Opioid Prescribing: Development and Testing of Draft Specifications | Syrowatka 2021 AMIA Annual Symposium Proceedings |  |  |  | electronic clinical quality measure  (eCQM)  to assess  potentially inappropriate high  dose post  operative opioid prescribing practices  e.g in this population an average daily dose  ≥90  morphine milligram equivalents  for the duration  of post operative opioid prescription in preoperatively opioid naïve patients |  |  |
| 27 | No Pain, More Gain: Reduced Postoperative  Opioid Consumption with a Standardized Opioid-  Sparing Multimodal Analgesia Protocol in Opioid-  Tolerant Patients Undergoing Colorectal Surgery | Truong 2019 The American Surgeon | Definition of ‘opioid tolerant’ made if any opioid in current meds on admission | opioid-sparing multimodal analgesia protocols developed and used to manage both opioid naïve and opioid tolerant patients | opioid-sparing multimodal analgesia protocols developed and used to manage both opioid naïve and opioid tolerant patients | opioid-sparing multimodal analgesia protocols developed and used to manage both opioid naïve and opioid tolerant patients |  |  |
| 28 | The Efficacy, Applicability and Side-Effects of  Postoperative Intravenous Patient-Controlled  Morphine Analgesia: An audit of 1233 Chinese patients | Tsui 1996 Anaesth Intens Care |  |  |  | Record rate of opioid associated side-effects:  Nausea, vomiting, dizziness, respiratory depression including bradypnoea  and oxygen desaturation to  less than 90% for longer than one minute. Any  required treatment with IV naloxone |  |  |
| 29 | One institution's experience with self-audit of opioid  prescribing practices for common cervical procedures | Wang 2021 Head and Neck |  |  |  |  | Procedure specific mean discharge MME  Discharge prescribing based on patient specific inpatient requirement  Procedure specific mean daily MME |  |
| 30 | Achieving major colorectal opioid free surgery: is it possible? | Yap 2019 Digestive Surgery | Presence of ERAS pathway including multimodal analgesia guideline and adherence to it | Presence of ERAS pathway including multimodal analgesia guideline and adherence to it | Presence of ERAS pathway including multimodal analgesia guideline and adherence to it | Presence of ERAS pathway including multimodal analgesia guideline and adherence to it |  |  |
| **Additional papers – references reviewed** |  |  |  |  |  |  |  |  |
| 31 | Opioids after hospital discharge from surgery | Bartels, 2016, PLOS one |  |  |  |  | Information and advice provided on how to store unused opioids (secure, locked location, dispose of back to pharmacy if unused) |  |
| 32 | New persistent opioid use after minor and major surgical procedures in US adults | Brummett, 2016, JAMA Surg | PPOU (ongoing opioid use at 90-180 days post op) is associated with risk factors pre-op; screen for these: Tobacco, alcohol and substance use pre op, mood disorders and anxiety and pre-op pain disorders. |  |  |  |  |  |
| 33 | Preoperative opioid use is independently associated with increased costs and worse outcomes after major abdominal surgery | Cron, 2017 Ann Surg | - Identify pre-operative opioid use in patients undergoing major abdominal surgery as it is associated with greater LOS and morbidity - Consider pre-op opioid use a modifiable risk factor |  |  |  |  |  |
| 34 | Opioid use after discharge in postoperative surgical patients | Hill,2017, Annals of Surgery |  |  |  |  | On discharge tailor opioid prescriptions to need to avoid unused opioid in the community.  Education on discharge on the need to dispose of unused opioid safely to avoid opioid diversion. |  |
| 35 | Post discharge opioid prescribing and use after common surgical procedures | Fujii, 2018, J Am Coll Surg |  |  |  |  | Protocolise discharge opioid prescribing (e.g using procedure-specific percentiles of MMEs) to limit inter-prescriber variation in amount prescribed on discharge  Provide education on safe storage and disposal of unused opioids |  |
| 36 | Enhanced Recovery Programme in Colorectal Surgery; A metaanalysis of randomised controlled trials | Greco, 2014, World J Surg |  |  |  | Protocolise use of opioids in hospital.  Avoid long acting opioids. |  |  |
| 37 | An educational intervention decreases opioid prescribing after general surgical operations | Hill, 2018, Annals of Surg |  |  |  |  | Develop operation-specific prescribing guidelines for surgeons | Define post-operative opioid requirements for patients using surveys |
| 38 | Prevalence of preoperative opioid use and characteristics associated with opioid use among patients presenting for surgery | Hilliard, 2018, JAMA Surg | Identify pre-op opioid use. Characteristics independently associated with pre-op opioid use are tobacco and illicit drug use, depression, higher Fibromyalgia scores, greater number of comorbidities  Recognition of preop opioid use as a complex population  Pre-op opportunity for weaning opioids and optimisation |  |  |  |  |  |
| 39 | Opioid related adverse events in post surgical pain control | Kessler, 2013, Pharmacotherapy |  |  |  | Define periop opioid-related adverse drug events (ORADEs). The presence of these increase LOS, 30-day mortality and morbidity.  Monitor and document nature and frequency of ORADEs  Eg GI related, GU related, respiratory related, CNS, other (eg bradycardia, itch).  Identify those at greatest risk of ORADEs. |  |  |
| 40 | New persistent opioid use among patients with cancer after curative-intent surgery | Lee 2017 J Clin Oncol | Consider ‘universal precautions’ for periop opioid use.  Opioid naïve patients:  Screen for those at risk of PPOU  Patient counselling and education on post-op pain, potential risks of opioids and need to minimise opioid use. |  |  |  | Use prescribing guidelines and avoid excessive opioid prescribing  Patient education on safe storage and disposal of opioids in the community |  |
| 41 | Costs and Consequences: A review of discharge opioid prescribing for ongoing management of acute pain | Macintyre, 2014, Anaesth Intensive Care | Screen for potential opioid misuse, abuse and diversion. Opioid Risk Tool (ORT) and Screener for Opioid Assessment and Patients with Pain (SOAPP) and Brief Risk Interview (BRI) may be of use in acute pain setting.  Adopt ‘Universal precautions’: screening for opioid abuse |  |  |  | Universal precautions:  Select appropriate opioid therapy.  Use prescription monitoring programme  Ensure discharge opioid matches need; surgical procedure and opioid requirement in the 24h prior to discharge  Identify those at risk of ORADEs when prescribing opioids for use at home. Male, obese, over 65, greater comorbidities, pre-op opioid use, concurrent sedative medication use.  Discharge patient advice: do not drive for up to 4 weeks until opioid dose is stable  Consider opioid type and duration of prescription on discharge  Employ ‘Reverse pain ladder’ as a de-escalation tool; strong opioid plus non-opioid, weak opioid plus non-opioid and finally non-opioid alone.  Avoid gaps in care in the transition from hospital to primary care. Pain management plan and tapering strategies must be clearly communicated to primary care team in a timely manner | Have a plan if opioid abuse or misuse is detected. |
| 42 | Adverse drug events among patients receiving postsurgical opioids in a large health system; risk factors and outcomes | Minkowitz, Am J Health Syst Pharm 2014 | Identify those at risk of ORADE (male, older age group, prior opioid use, diverticulitis, ulcerative colitis, COPD, cardiac dysrhythmia) |  |  |  |  |  |
| 43 | Inappropriate opioid prescription after surgery | Neuman2019 The Lancet | Patient education on expectations of pain control and risks and benefits of opioid use | Consider use of opioid sparing or opioid free anaesthesia and analgesia in context of ERAS programme |  | Use local guidelines for opioid prescribing for common procedures | Match opioid prescription to requirement – number of pills and duration  Use an algorithm to individualise opioid prescribing after discharge. Relate this to opioid use in the 24h prior to discharge  Patient education on safe opioid storage and disposal |  |
| 44 | What factors are associated with increased risk for prolonged postoperative opioid usage after colorectal surgery? | Stafford, 2018, Surgical Endoscopy | Screen patients at risk of PPOU (younger age, history of chronic pain, depression, history of substance abuse). | Use of minimally invasive techniques associated with reduced risk of long term opioid use |  |  |  |  |
| 45 | Standardisation of care in colorectal surgery | Thiele 2015 J Am Coll Surg |  | Reduce opioid use through regional blocks and multimodal analgesia as part of a protocol | Reduce opioids by use of protocolised multimodal analgesia | Reduce opioids by use of protocolised multimodal analgesia |  |  |
| 46 | Wide variation and overprescription of opioids after elective surgery | Thiels, 2017 Annals of Surgery |  |  |  |  | Protocolise number of pills and duration of discharge opioid prescription for common procedures to reduce overprescription and inter-prescriber variation |  |
| 470-60 | Postoperative multimodal analgesia pain management with non-opioid analgesics and techniques; a review | Wick 2017 JAMA Surgery |  | Use protocols to guide multimodal analgesia and techniques to limit intra op opioid delivery | Use protocols to guide multimodal analgesia and techniques to limit post op opioid delivery | Use protocols to guide multimodal analgesia and techniques to limit post op opioid delivery | Use protocols to guide multimodal analgesia and techniques to limit discharge opioid requirement |  |
|  | **Paper** | **Reference**  **(1^st^ author, year of publication)** | **Pre admission/ pre op** | **Intra**  **op** | **Recovery** | **Post op** | **Discharge** | **Follow up** |
