# Supplementary material for: Process, structural, and outcome quality indicators to support perioperative opioid stewardship: a rapid review
Source: Perioper Med (Lond). 2023 Jul 10;12:34. doi: 10.1186/s13741-023-00312-4 (PMC10332041; doi:10.1186/s13741-023-00312-4)
Supplement: Supplementary file 2 — Additional file 2: Supplementary materials 2. Characteristics of papers [file 13741_2023_312_MOESM2_ESM.docx]

Appendix 2: The characteristics of included papers

| **First author, year of publication** | **Country** | **Setting/ n participants** | **Type of study** | **Aim of study** | **Quality of Study** |
| --- | --- | --- | --- | --- | --- |
| Agarwal, 2020 | USA | 2 hospitals, 919 participants | Quality improvement | To assess the difference between the number of opioid tablets prescribed and the self-reported number of tablets taken with use of an automated text messaging system | Unable to score; quality improvement project |
| Bardiau, 1999 | Belgium | 1 hospital, 1975 participants | Open, prospective, nonrandomized observational study | To assess the effects of an Acute Pain Service inception on postoperative pain management in a general teaching hospital using pain indicators as performance measures | Newcastle-Ottowa Scale: intermediate quality  Selection ****  Comparability  Outcome ** |
| Bartels, 2016 | USA | 1 hospital, 64 participants | Prospective cohort | To investigate opioid use and storage patterns by patients following hospital discharge following surgery | Newcastle-Ottowa Scale: intermediate quality |
| Bongiovanni, 2020 | USA | 1 hospital, 262 participants | Quality improvement | To investigate whether a trainee-led quality improvement programme can lead to an increase in the percentage of patients discharges with non-opioid analgesic medication | Unable to score; quality improvement project |
| Brandal 2017 | USA | 1 hospital, 383 participants | Historic-prospective quality improvement | To assess the impact of  an ERAS intervention for colorectal surgery on discharge opioid prescribing practices |  |
| Brat, 2018 | USA | De-identified administrative database (multicentre), 1015116 participants | Retrospective cohort study | To quantify the effects of varying opioid prescribing patterns after surgery on dependence, overdose, or abuse in an opioid naive population |  |
| Bromberg, 2021 | USA | 1 hospital, 304 participants | Prospective cohort | To evaluate the prescribing practices and opioid consumption in an ambulatory setting to inform development of evidence based guidelines |  |
| Brummett, 2017 | USA | National insurance claims dataset (multicentre), 36177 participants | Retrospective cohort | To determine the incidence of new and persistent opioid use after minor and major surgical procedures |  |
| Chen, 2018 | USA | 3 hospitals, 18434 participants | Retrospective, cross- sectional | To determine correlation between a post operative patient’s 24h pre-discharge opioid use and the amount of opioids prescribed at hospital discharge |  |
| Cheung, 2008 | Hong Kong | 1 hospital, 5137 participants | Retrospective cohort | To investigate the effects of an acute pain service and patient-controlled analgesia on pain control and side effects |  |
| Clarke, 2014 | USA | State-wide administrative database (multicentre), 39140 participants | Population based retrospective cohort study | To describe rates and risk factors for prolonged postoperative  use of opioids in patients who had not previously used opioids and  undergoing major elective surgery |  |
| Cron 2017 | USA | 1 hospital, 2413 participants | Retrospective cohort | To explore the clinical and financial implications of preoperative opioid use in major abdominal surgery |  |
| Felling, 2018 | USA | 1 hospital, 179 participants | Single institution, open-label  randomized (1:1) trial | To compare  liposomal bupivacaine transversus abdominis plane block  with epidural analgesia in patients undergoing colorectal surgery |  |
| Fields, 2019 | USA | 5 hospitals, 1243 participants | Retrospective cohort | To determine the rate of and risk factors for prolonged opioid use following colectomy |  |
| Fujii, 2018 | USA | 2 hospitals, 10471 participants | Retrospective dataset and prospective cohort study | To identify opioid prescribing and use patterns after surgery to inform evidence based practices |  |
| Gan, 2015 | USA | Research database (multicentre), 138068 participants | Retrospective cohort | To assess the incidence and economic impact of postoperative ileus following laparotomy and  laparoscopic procedures for colectomies and cholecystectomies in patients receiving postoperative pain management with opioids |  |
| Gan, 2020 | USA | NSQIP database (multicentre), 1201 participants | Retrospective cohort | To evaluate the impact of preoperative prescription opioid, sedative, and antidepressant use on postoperative outcomes following colorectal surgery |  |
| Greco, 2014 |  | 16 RCTs, 2376 participants | Meta-analysis of RCTs | To assess the impact of the ERAS pathway on morbidity, length of stay and readmission rate following colorectal surgery | 16 RCTs assessed using Cochrane risk of bias tool:  7 low risk of bias, 4 intermediate risk of bias, 5 high risk of bias. |
| Hill, 2017 | USA | 642 patients | Prospective cohort | To examine opioid prescribing practices and to determine optimal number of pills to prescribe | Newcastle-Ottowa Scale: intermediate quality |
| Hill, 2018 | USA | 1 hospital, 246 patients | Prospective cohort | To determine whether an educational intervention is sufficient to decrease opioid prescribing after general surgical operations |  |
| Hilliard, 2018 | USA | 1 hospital, 34186 participants | Retrospective cohort | To assess the prevalence of preoperative opioid use and the characteristics of these patients |  |
| Hoang, 2020 | USA | 1 hospital, 443 participants | Retrospective cohort | To identify the changes in opioid-  prescribing patterns of colorectal surgical practice as a result of legislative change |  |
| Hopkins, 2020 | Australia | 1 hospital, 4062 participants | Cluster randomized controlled trial | To evaluate whether educating junior doctors and hospital pharmacists about analgesic prescribing improved discharge prescribing of opioids for opioid- naïve patients after surgical admissions |  |
| Howard, 2019 | USA | 33 hospitals, 2392 participants | Retrospective cohort | To describe opioid prescribing and consumption for a variety of surgical procedures and to determine factors associated with opioid consumption after surgery |  |
| Jiang, 2017 | USA | Electronic database (multicentre), 79123 participants | Retrospective, cross sectional | To investigate the prevalence and disparity of chronic opioid usage in surgical patients and the potential risk factors associated with chronic opioid usage |  |
| Keller, 2019 | USA | Research database (multicentre), 50098 procedures | Retrospective cohort | To evaluate the impact of opioid utilization on quality measures and costs after open and laparoscopic colorectal surgery |  |
| Kessler, 2012 | USA | 26 hospitals, 37031 participants | Retrospective cohort | To determine the prevalence of inpatient opioid use in the postoperative setting and to investigate frequency and risk factors for opioid related adverse drug events, ORADEs. |  |
| Lee, 2010 | Hong Kong | 1 hospital, 423 participants | Randomised controlled trial | To investigate the costs and effects of acute pain service care on clinical outcomes after major elective surgery |  |
| Lee, 2017 | USA | National insurance claims dataset (multicentre), 68463 participants | Retrospective cohort | To define the risk of new, persistent opioid use after curative-intent cancer surgery |  |
| Macintyre, 2014 |  |  | Review article | To review discharge opioid prescribing for the ongoing management of acute pain after surgery |  |
| Meyer, 2021 | USA | 1 hospital, 174 patients | Retrospective cohort | To define opioid consumption patterns following anorectal operations for development of an institutional prescribing guideline |  |
| Minkowitz, 2014 | USA | 11 hospitals, 6285 participants | Retrospective cohort | To investigate the effect of opioid related adverse drug events in the postsurgical population |  |
| Mujukian, 2019 | USA | 1 hospital, 71 participants | Prospective cohort study | To investigate the effect of a multimodal analgesia protocol on opioid consumption perioperatively in patients undergoing minimally invasive colorectal surgery |  |
| Neuman, 2019 |  |  | Review article | To review inappropriate opioid prescription after surgery |  |
| Oderda, 2013 | USA | Electronic health record (multicentre), 319 898 procedures | Retrospective cohort | To investigate the incidence of opioid-related adverse drug events (ORADEs), and the impact of ORADEs on patient outcomes and health economics |  |
| Pruitt, 2019 | USA | 23 hospitals, 2239 participants | Quality improvement | To assess and reduce the overprescription of opioids to patients undergoing general surgical procedures |  |
| Pullman, 2021 | USA | Electronic health record (multicentre), 12803 participants | Retrospective cohort | To design and implement an electronic clinical quality measure (eCQM) assessing the rate of prolonged opioid prescribing  practices following total hip arthroplasty and total knee arthroplasty |  |
| Roughead, 2019 | Australia | Administrative health claims database (multicentre), 24854 participants | Retrospective cohort | To determine time to opioid cessation post  discharge from hospital in persons who had been admitted to hospital for a surgical procedure and were previously naïve to opioids |  |
| Stafford, 2018 | USA | 1 hospital, 9423 participants | Retrospective cohort | To investigate factors associated with prolonged postoperative opioid use after colorectal surgery |  |
| Syrowatka, 2021 | USA | Electronic health record, (multicentre), 9108 participants | Retrospective cohort | To re-tool an existing claims based measure, ‘Use of Opioids at High Dosage in Persons Without Cancer’ to an electronic clinical quality measure to assess potentially inappropriate high-dose postoperative opioid prescribing practices |  |
| Thiele, 2015 | USA | 1 hospital, 207 participants | Before and after study design | To investigate the effect of an enhanced recovery programme on length of stay, complications and costs after colorectal surgery |  |
| Thiels, 2017 | USA | 3 hospitals, 7651 participants | Retrospective cohort | To identify opioid prescribing practices across surgical specialties and institutions |  |
| Truong, 2019 | USA | 1 hospital, 201 participants | Retrospective cohort | To determine the impact of a standardized opioid sparing multimodal analgesia protocol in opioid tolerant vs opioid naïve patients after major colorectal surgery |  |
| Tsui, 1996 | Hong Kong | 1 hospital, 1233 participants | Retrospective cohort | To investigate efficacy and side effects of morphine patient-controlled analgesia after major surgery |  |
| Wang, 2021 | USA | 1 hospital, 472 participants | Retrospective cohort | To investigate opioid prescribing practices for inpatients v discharge prescription |  |
| Wick, 2017 |  | 27 RCTs, 2314 patients | Meta-analysis | To define important aspects of opioid sparing analgesic regimes |  |
| Yap 2021 | Australia | 1 hospital, | Retrospective cohort | To investigate perioperative opioid usage with an opioid sparing enhanced recovery protocol and to identify factors contributing to opioid free surgery |  |
