# Supplementary material for: Process, structural, and outcome quality indicators to support perioperative opioid stewardship: a rapid review
Source: Perioper Med (Lond). 2023 Jul 10;12:34. doi: 10.1186/s13741-023-00312-4 (PMC10332041; doi:10.1186/s13741-023-00312-4)
Supplement: Supplementary file 3 — Additional file 3: Supplementary materials 3. Quality indicators and themes [file 13741_2023_312_MOESM3_ESM.docx]

| Appendix 3: Extracted quality indicators grouped into themes | |
| --- | --- |
| Theme | Extracted quality indicators |
| Patient education | The provision of patient education materials preoperatively on expectations of perioperative pain and pain management options including the risks and benefits of opioids ^28, 42, 56, 57, 61^ |
|  | The provision of patient education materials on discharge on safe storage and disposal of unused opioids in the community and the requirement to avoid opioid diversion ^29,40, 43^, and opioid specific discharge advice, e.g. DVLA requirements ^57^ |
| Staff education | The provision of multi-professional education materials on opioid stewardship ^31,50^ |
|  | The provision of multi-professional education materials on the provision of multimodal analgesia at all stages of the patient journey starting in the preoperative setting ^31,50^ |
|  | Prescribers receiving quarterly reports comparing their prescribing to hospital guidelines ^63^ |
|  | Education on the need for the pain management plan and tapering strategies to be clearly communicated to primary care team in a timely manner on discharge ^57^ |
| Preoperative patient optimisation | The provision of a specialist pain service and referral pathway to enable opioid weaning and patient-specific analgesic planning for preoperative optimization for patients with opioid tolerance ^42, 48^ |
|  | The provision of an individualised prescribing plan for patients with opioid tolerance ^48^ |
|  | The presence of a system to allow the identification of preoperative opioid use in the elective population ^33, 39, 42, 45, 48, 70^ |
|  | The use of a preoperative screening tool to identify patients with risk factors risk of persistent postoperative opioid use ^35,38, 42, 52, 56, 57, 66^ |
| Patient and procedure- specific prescribing and deprescribing | The availability of an acute pain service ^28^ |
|  | The availability of a daily pain review ^32^ |
|  | Use of the electronic record as a means to detect or highlight potentially inappropriate high-dose postoperative opioid prescriptions ^63,^ ^67^ |
|  | The presence of and adherence to a perioperative analgesia protocol which includes regional blocks and multimodal analgesia ^28, 32,^ ^37, 41, 53, 60, 61, 66, 68, 70, 73, 74^ |
|  | The presence of and adherence to procedure-specific protocols for use of in-hospital opioids specifically promoting the avoidance of long acting opioids ^32,^ ^46 ,56^ |
|  | The presence of review postoperatively with new risk factors for PPOU identified including e.g. formation of a stoma ^42^ |
|  | On discharge, the use of the electronic record to enable procedure-specific prescribing limits ^63, 67^ |
|  | The use of protocolized opioid prescribing for hospital discharge:  The use of procedure-specific Milligram of Morphine Equivalent (MME) centiles to guide prescribing ^33,^ ^43, 63, 72^  The presence of a process to review the procedure-specific mean discharge MME prescribed for that patient group ^29,49, 57^  The presence of a patient group-specific guideline or algorithm for discharge opioid prescribing ^32,^ ^40,^ ^42,^ ^47, 56, 57, 61, 69^  The use of procedure -specific postoperative prescribing guidelines to provide enough doses to cover 75% of patients ^63^  The use of the opioid requirement in the 24h leading up to discharge as a guide for opioids prescribed on discharge ^36, 57, 61, 72^ |
|  | The total MME consumed during the 24 hours prior to discharge ^32, 57, 61, 72^  The total MME consumed during the entire hospital stay ^32^  The procedure-specific mean daily inpatient MME used ^50,^ ^51, 72^  Use of higher dosage of opioids at any time (>50-60 MME) used to flag need for review ^33,^ ^42^ |
|  | The presence of a review process for opioid prescription at discharge:  The presence of opioids on the hospital discharge prescription ^32, 50^  The frequency of slow-release opioid prescription on discharge ^50^  The frequency of immediate-release opioid prescription on discharge ^50^  The frequency of non-opioid adjuvant analgesia prescription on discharge ^50^ |
|  | The presence of a de-escalation plan for opioids prescribed on discharge ^50,^ ^57^  Protocolised use of the ‘reverse pain ladder’ to guide de-escalation  Pain management plan and tapering strategy clearly communicated to the primary care team in a timely manner ^57^ |
|  | The presence of a process to assess opioids prescribed versus opioids actually used following surgical procedures to allow tailoring of opioid prescriptions to need for a patient group/specific procedure ^27, 29, 34, 40, 47, 51, 58, 61, 63^ |
|  | The presence of patient screening for risk of PPOU at discharge ^42, 65^  Follow up for patients at greatest risk of persistent postoperative opioid use ^42^ |
|  | Detection of new or repeat opioid prescriptions given within 30 days of discharge ^33,^ ^49^ |
|  | The presence of a protocol or clear plan to follow if opioid abuse or misuse is detected ^57^ |
|  | The incidence of opioid-related re-admissions ^42^ |
|  | The incidence of patients still using opioids at 90-180 days postoperatively ^33,38, 64, 65^ |
| Opioid-related adverse drug events (ORADEs) | The presence of a preoperative screening tool to identify patients at risk of postoperative opioid related adverse drug events (ORADEs) ^53, 54, 57, 59,62^ |
|  | The presence of a system to detect and score ORADEs amongst postoperative inpatients ^53, 54, 55, 62, 71^ |
|  | The presence of a system to detect ORADEs in the community setting following discharge ^54^ |
